# The additive effects of kidney dysfunction on left ventricular function and strain in type 2 diabetes mellitus patients verified by cardiac magnetic resonance imaging

**DOI:** 10.1186/s12933-020-01203-4

**Published:** 2021-01-07

**Authors:** Yi Zhang, Jin Wang, Yan Ren, Wei-feng Yan, Li Jiang, Yuan Li, Zhi-gang Yang

**Affiliations:** 1 Department of Radiology, National Key Laboratory of Biotherapy, West China Hospital, Sichuan University, 37# Guo Xue Xiang, Chengdu, 610041 Sichuan China; 2 Department of Endocrinology and Metabolism, West China Hospital, Sichuan University, 37# Guo Xue Xiang, Chengdu, 610041 Sichuan China

**Keywords:** Type 2 diabetes mellitus, Chronic kidney disease, Left ventricular, Strain, Magnetic resonance imaging

## Abstract

**Background:**

Patients with type 2 diabetes mellitus (T2DM) are susceptible to coexisted with chronic kidney disease (CKD), which may increase cardiovascular mortality in these patients. The present study aimed to verify whether CKD aggravates the deterioration of left ventricular (LV) myocardial strain in T2DM patients and to explore the risk factors associated with LV strain.

**Materials and methods:**

In total, 105 T2DM patients and 52 healthy individuals were included and underwent cardiac magnetic resonance examination. Patients were divided into the following two groups: T2DM with CKD (n = 33) and T2DM without CKD (n = 72). The baseline clinical and biochemical indices were obtained from hospital records before the cardiac magnetic resonance scan. Cine sequences, including long-axis views (2-chamber and 4-chamber) and short-axis views, were acquired. LV function and global strain parameters were measured based on cine sequences and compared among three groups. Pearson’s analysis was performed to investigate the correlation between LV strain parameters and clinical indices. Multiple linear regression analysis was used to identify the independent indicators of LV strain.

**Results:**

Compared with normal controls, T2DM patients without CKD had a significantly decreased magnitude of peak strain (PS; radial), peak systolic strain rate (radial), and peak diastolic strain rate (radial and circumferential) (all P < 0.05). Furthermore, T2DM patients with CKD displayed markedly lower magnitudes of PS (radial, circumferential, and longitudinal) and peak diastolic strain rate (circumferential and longitudinal) than both normal controls and T2DM patients without CKD (all *P* < 0.05). The eGFR was positively associated with the magnitude of PS (R = radial, 0.392; circumferential, 0.436; longitudinal, 0.556), while uric acid was negatively associated with the magnitude of PS (R = radial, − 0.361; circumferential, − 0.391; longitudinal, − 0.460) (all P < 0.001). Multivariable linear regression indicated that the magnitude of PS was independently associated with eGFR (β = radial, 0.314; circumferential, 0.292; longitudinal, 0.500) and uric acid (β = radial, − 0.239; circumferential, − 0.211; longitudinal, − 0.238) (all P < 0.05).

**Conclusions:**

Kidney dysfunction may aggravate the deterioration of LV strain in T2DM patients. LV strain is positively associated with the estimated glomerular filtration rate and negatively associated with uric acid, which may be independent risk factors for predicting reduction of LV strain.

## Background

Diabetes mellitus (DM) has caused a severe public health burden with an estimated 451 million people living with diabetes worldwide [1]. A common comorbidity of DM, especially type 2 diabetes diabetes mellitus (T2DM), is chronic kidney disease (CKD), which is associated with an increased risk of cardiovascular disease, heart failure, infections, reduced quality of life, increase of financial burden and premature mortality [2, 3]. In DM patients with CKD, high glucose concentrations and hyperfiltration lead to extracellular matrix deposition, oxidative stress, chronic inflammation, hypertrophy, and neuronal abnormalities resulting in stiffening of the myocardium [4]. It has been demonstrated that cardiovascular morbidity and mortality in DM patients with CKD are substantially increased compared to patients with DM alone. [1]. These observations underscore the importance of evaluating cardiac function in T2DM patients with CKD to mitigate CVD risk and improve outcomes.

Cardiac magnetic resonance (CMR) have served as the gold standard modality to assess cardiac structure and function. CMR derived strain based on cine sequence and tissue-tracking techniques has been proven to provide quantitative assessment of myocardial deformation based on every voxel of cine sequence [5–8]. Relationships between CMR indices and metabolic-associated factors (e.g., duration of diabetes, obesity, hypertension, and dyslipidaemia) have been investigated in uncomplicated DM patients in several previous studies [7–10]. However, to the best of our knowledge, studies focusing on the combined effect of T2DM with CKD on cardiac function are scarce. Therefore, this study aimed to verify whether kidney dysfunction aggravates the deterioration of left ventricular (LV) myocardial strain in diabetic patients and to investigate the association between LV strain and kidney function indices.

## Materials and methods

### Study population

A total of 138 patients with clinically diagnosed T2DM (based on the current American Diabetes Association guidelines) were prospectively recruited in the present study between September 2016 and September 2020 [11]. Diabetic patients with CKD are clinically defined by the presence of persistent low eGFR (< 60 mL/min/1.73 m^2^) [12]. We excluded individuals with primary or secondary cardiomyopathy diseases not induced by DM (n = 24) [13], patients with contraindications to CMR imaging (n = 4), and patients with poor quality CMR images due to arrhythmia (n = 5). Finally, 105 remaining patients participated in the study and received the CMR examination. Concurrently, 52 age- and sex-matched healthy volunteers with no history of impaired glucose tolerance; known systematic disease such as hypertension, hyper-lipidemia; electrocardiogram abnormalities; and cardiovascular abnormalities detected by CMR (abnormal ventricular motion, reduced LVEF, valvular stenosis) were included as normal controls.

Data on demographic characteristics, including age, gender, height, weight, systolic/diastolic blood pressure, and resting heart rate, were collected at the time of CMR scanning. The body mass index (BMI) was calculated as weight (kg) divided by the square of height (m). Blood pressure and resting heart rate were recorded as an average of three measurements in the right arm in a sitting position after a 10-min resting period. Data regarding diabetes duration and biochemical indices, including fasting plasma glucose, glycated hemoglobin (HbA1c), total cholesterol, triglycerides, high density lipoprotein (HDL), low density lipoprotein (LDL), creatinine, urea, and uric acid were obtained from hospital records before the CMR examination. The estimated glomerular filtration rate (eGFR) was calculated by creatinine using the CKD-EPI equation [14]. In addition, related complications (retinopathy, neuropathy, peripheral vascular disease, atrial fibrillation) and medications (Insulin, Biguanides, Sulfonylureas, α-Glucosidase inhibitor, Glucagon-like peptide-1/Dipeptidyl peptidase-4 inhibitor, Sodium-glucose co-transporter 2 inhibitor, Statin, Angiotensin-converting enzyme inhibitor and/or Angiotensin receptor blocker, β-blockers, Calcium channel blocker, Loop diuretics, Spironolactone, Thiazides) were also collected from hospital records.

### CMR protocol

All participants underwent CMR examination in the supine position using a 3.0T whole-body scanner (Skyra; Siemens Medical Solutions, Erlangen, Germany). A manufacturer’s standard ECG-triggering device and the breath-hold technique were used to monitor the dynamic changes of ECG and breathing, respectively. Data acquisition was performed during the breath-holding period at the end of inspiration. A series of cine sequences, including 8–12 continuous short-axis views of LV from the mitral valve to the level of the LV apex, were obtained using steady-state free precession (repetition time, 33.22 ms; echo time, 1.31 ms; flip angle, 39°; slice thickness, 8.0 mm; field of view, 234 × 280 mm^2^; and matrix size, 208 × 139 pixels) as well as the horizontal 4-chamber and vertical 2-chamber long-axis. LV function parameters and tissue-tracking indices were assessed from these continuous cine sequences. The condition of the participants remained stable throughout the examination.

### Image analysis

Image analysis was performed by two experienced radiologists with more than 3 years of CMR experience using commercial software (cvi42; Circle Cardiovascular Imaging, Inc., Calgary, AB, Canada). The endocardial and epicardial contours were delineated manually per slice at the end-diastole and end-systole, and the papillary muscles and moderator bands were carefully excluded in all series. The global LV geometry and function parameters, including end-diastolic volume (EDV), end-systolic volume (ESV), systolic volume (SV), LV ejection fraction (LVEF), and LV mass, were automatically computed according to the current guidelines [15]. In addition, the LV remodelling index, calculated as LV mass divided by LVEDV, was included for analysis [16]. Global LV strain variables were analysed based on tracking every voxel of myocardium on short-axis, horizontal 4-chamber long-axis, and vertical 2-chamber long-axis cine slices.

The strain variables included peak strain (PS, defined as the absolute highest value of the strain over all phases of the cardiac cycle), peak systolic strain rate (PSSR, defined as the absolute highest value of the strain rate starting from a diastole to the next systole), and peak diastolic strain rate (PDSR, defined as the maximum absolute value of the strain rate starting from a systole to the next diastole) in radial, circumferential, and longitudinal directions. The radial direction was perpendicular to the circumferential direction towards the centroid of the left ventricle. The circumferential direction was parallel to the tangent of the epicardial surface. The longitudinal direction was perpendicular to the radial axis pointing from the base to the apex of the ventricle. Radial strain was considered positive due to the thickening of the myocardium when the left ventricle was contracting. Circumferential and longitudinal strains were considered negative because the myocardium shortened when contracting [17].

### Reproducibility

The intraobserver variability in the LV global strain parameters was assessed by an experienced investigator (YZ) by comparing the measurements from 60 randomly selected cases analysed by the same observer after one month. The interobserver variability was evaluated by comparing the measurements from the same population by another independent double-blinded experienced observer (JW).

### Statistical analyses

All statistical analyses were performed using SPSS software (Version 21.0, Armonk, New York, USA), and statistical diagrams were plotted by GraphPad Prism software (version 7.0a, GraphPad Software Inc., San Diego, CA, USA). The Kolmogorov-Smirnov test was performed to check for normality of continuous variables. Data are presented as the mean ± standard deviation for variables with continuous normal distribution and median (25–75% interquartile range) for those with continuous non-normal distribution. Comparisons for normally distributed variables were performed by one-way analysis of variance with the Bonferroni post-hoc correction among normal controls and T2DM patients with and without CKD. The kidney function indices were compared between T2DM patients with and without CKD using Student’s t-test. The Kruskal-Wallis rank test was performed to evaluate categorical variables and parameters that did not conform to normality or homogeneity of variance. Spearman’s test was used for correlation analysis between PS and clinical indices (e.g., creatinine, eGFR, urea, uric acid, diabetes duration, fasting plasma glucose, HbA1c, total cholesterol and triglycerides). Furthermore, variables with a probability value of < 0.1 and the absence of collinearity were included in a multiple linear regression analysis adjusting for age, gender, BMI, systolic blood pressure, rest heart rate to identify the independent indicators reflecting the severity of LV PS. The intraclass correlation coefficient (ICC) was used to evaluate both inter- and intraobserver variability. All statistical calculations followed a two-tailed test, and a *P*-value of < 0.05 was considered statistically significant.

## Results

### Participant characteristics

In total, 157 participants (105 patients and 52 controls) were included in the present study. Of all 105 diabetic patients, 33 (31.4%) diabetic patients were diagnosed with CKD, and 72 (68.6%) diabetic patients were diagnosed without CKD. Table 1 presents the baseline characteristics, diabetic status, lipid status, kidney function indices, complications, and medications. Subjects in the three groups were similar in age, gender proportion, and BMI. The T2DM with CKD group showed markedly higher creatinine [142.6 (121.0–200.0) µmol/L vs. 70.3 ± 15.5 µmol/L], urea [10.0 (7.2–12.3) mmol/L vs. 5.7 (4.7–6.8) mmol/L], and uric acid (479.0 ± 154.4 µmol/L vs. 321.7 ± 105.8 µmol/L) levels but lower eGFR (38.2 ± 14.8 ml/min/1.732 m^2^ vs. 96.1 ± 14.5 ml/min/1.732 m^2^) levels than those of the T2DM without CKD group (all P < 0.05).

**Table 1:** Baseline characteristics of the study cohort

	Controls (n = 52)	T2DM patients	
		Without CKD (n = 72)	With CKD (n = 33)
Male, n (%)	36 (69.2)	50 (69.4)	23 (69.7)
Age, years	56.4 ± 9.9	57.1 ± 8.5	56.9 ± 15.7
BMI, kg/m^2^	22.5 (20.5–24.5)	23.9 ± 2.7	24.5 ± 3.4
Systolic blood pressure, mmHg	119.2 ± 5.3	125.5 (116.0–130.0)	132.6 ± 30.9^a,b^
Diastolic blood pressure, mmHg	80.0 (75.0–83.0)	78.7 ± 10.0	80.2 ± 18.2
Rest Heart rate, bmp	74.2 ± 9.9	73.6 ± 11.2	87.8 ± 11.2^a,b^
Diabetes duration, years	–	6.0 (3.0–9.9)	10.0 ± 5.3^b^
Diabetic status			
Fasting plasma glucose, mmol/L	–	9.2 (7.3–11.0)	7.4 (6.4–12.0)
HbA1c, %	–	7.6 (6.6–9.0)	7.9 ± 1.2
Lipid status			
Total cholesterol, mmol/L	–	4.3 (3.6–5.1)	4.3 (3.5–5.2)
Triglycerides, mmol/L	–	1.4 (1.0-2.2)	1.4 (1.2–2.3)
HDL, mmol/L	–	1.1 (0.9–1.4)	1.0 ± 0.4^b^
LDL, mmol/L	–	2.4 ± 0.8	3.1 ± 2.1^b^
Kidney function indices			
Creatinine, umol/L	–	70.3 ± 15.5	142.6 (121.0-200.0)^b^
eGFR, ml/min/1.732 m^2^	–	96.1 ± 14.5	38.2 ± 14.8^b^
Urea, mmol/L	–	5.7 (4.7–6.8)	10.0 (7.2–12.3)^b^
Uric acid, umol/L	–	321.7 ± 105.8	479.0 ± 154.4^b^
Complications, n (%)			
Retinopathy	–	4 (5.6)	4 (12.1)
Neuropathy	–	4 (5.6)	5 (15.2)
Peripheral vascular disease	–	2 (2.8)	5 (15.2)^b^
Atrial fibrillation	–	4 (5.6)	3 (9.1)
Medications, n (%)			
Insulin	–	20 (27.8)	18 (54.5)^b^
Biguanides	–	41 (56.9)	19 (57.6)
Sulfonylureas	–	3 (3.9)	3 (9.1)
α-Glucosidase inhibitor	–	18 (25.0)	9 (27.3)
GLP-1/DPP-4 inhibitor	–	5 (6.9)	4 (12.1)
SGLT2 inhibitor	–	10 (13.9)	5 (15.2)
Statin	–	13 (18.1)	17 (51.5)^b^
ACEI and/or ARB	–	12 (16.7)	8 (24.2)
β-blockers	–	12(16.7)	14 (42.4)^b^
Calcium channel blocker	–	7 (9.7)	10 (13.9)^b^
Loop diuretics	–	5 (6.9)	14 (19.4)^b^
Spironolactone	–	5 (6.9)	13 (18.1)^b^
Thiazides	–	2 (2.8)	2 (2.8)

^a^T2DM patients vs. controls (*P* < 0.05)

^b^T2DM patients with CKD vs. T2DM patients without CKD (*P* < 0.05)

*T2DM *type 2 diabetes diabetes mellitus, *CKD *chronic kidney disease, *BMI *body mass index, *HDL *high-density lipoprotein, *LDL *low-density lipoprotein, *eGFR *estimated Glomerular Filtration Rate, *HbA1c *glycated hemoglobin, *GLP-1 *glucagon-like peptide-1, *DPP-4 *dipeptidyl peptidase-4, *SGLT2 *sodium-glucose co-transporter 2, *ACEI *angiotensin-converting enzyme inhibitor, *ARB *angiotensin receptor blocker

### Comparison of LV function and strain among T2DM groups and normal individuals

By comparison of LV function parameters, the T2DM without CKD group exhibited an increase in LVESV (62.48 ± 55.31 mL vs. 44.86 ± 12.41 mL), LV mass (96.14 ± 34.18 g vs. 72.14 ± 19.12 g), and LV remodelling index (0.73 ± 0.20 g/mL vs. 0.58 ± 0.13 g/mL) compared to normal controls (all P < 0.05). However, there was no significant difference in LVSV and LVEF between the T2DM without CKD group and the normal group (P = 0.076).

By comparison of LV strain parameters, the T2DM without CKD group had significantly lower radial PS compared to normal individuals (30.82 ± 10.48% vs. 38.42 ± 8.73%, *P* < 0.05). Regarding systolic function, only radial PSSR was reduced in the T2DM without CKD group compared to the normal group (1.67 ± 1.07 1/s vs. 2.14 ± 0.65 1/s) (*P* < 0.05). There was no significant difference in circumferential and longitudinal PSSR between T2DM groups and normal individuals. Regarding diastolic function, the magnitude of radial PDSR (– 1.72 ± 1.74 1/s vs. – 2.80 ± 0.91 1/s) and circumferential PDSR (1.10 ± 0.39 1/s vs. 1.25 ± 0.27 1/s) decreased in T2DM patients compared to the normal group (all *P* < 0.05) (Table 2).

**Table 2:** Comparison of CMR findings among T2DM patients with/without CKD and normal controls

	Controls (n = 52)	T2DM patients	
		Without CKD (n = 72)	With CKD (n = 33)
LV function parameters			
LVEDV, mL	121.09 ± 27.13	139.04 ± 61.75	161.04 (112.09–209.46)^a^
LVESV, mL	44.86 ± 12.41	62.48 ± 55.31^a^	75.90 (42.13–143.34)^a^
LVSV, mL	76.40 ± 18.56	76.55 ± 23.96	75.89 ± 36.97
LVEF, %	62.62 ± 5.80	58.45 ± 14.14	47.94 (31.33–65.10)^a,b^
LV mass, g	72.14 ± 19.12	96.14 ± 34.18^a^	124.02 ± 49.32^a,b^
LV remodelling index, g/mL	0.58 ± 0.13	0.73 ± 0.20^a^	0.77 ± 0.29^a^
Peak strain(%)			
Radial	38.42 ± 8.73	30.82 ± 10.48^a^	20.79 ± 12.02^a,b^
Circumferential	– 19.94 ± 2.71	– 19.37 ± 5.48	– 13.48 ± 6.16^a,b^
Longitudinal	– 14.82 ± 3.33	– 13.49 ± 3.78	– 8.35 ± 3.39^a,b^
PSSR(1/s)			
Radial	2.14 ± 0.65	1.67 ± 1.07^a^	1.28 ± 0.73^a^
Circumferential	– 0.97 ± 0.37	– 0.95 ± 0.48	– 0.82 ± 0.38
Longitudinal	– 0.76 ± 0.44	– 0.76 ± 0.70	– 0.61 ± 0.28
PDSR(1/s)			
Radial	– 2.80 ± 0.91	– 1.72 ± 1.74^a^	– 1.10 ± 0.90^a^
Circumferential	1.25 ± 0.27	1.10 ± 0.39^a^	0.80 ± 0.35^a,b^
Longitudinal	0.94 ± 0.28	0.95 ± 0.65	0.62 ± 0.26^a,b^

^a^T2DM patients vs. controls (*P* < 0.05)

^b^T2DM patients with CKD vs. T2DM patients without CKD (*P* < 0.05)

*T2DM *type 2 diabetes diabetes mellitus, *LV *left ventricular, *EDV *end diastolic volume, *ESV *end systolic volume, *SV *stroke volume, *EF *ejection fraction, *PSSR *peak systolic strain rate, *PDSR *peak diastolic strain rate

### Progressive deterioration of LV function and strain in T2DM patients with CKD

The T2DM with CKD group showed significantly higher values for LVEDV, LVESV, LV mass, and LV remodelling index but lower values for LVEF compared to normal controls (all *P* < 0.05). Moreover, compared to the T2DM without CKD group, LVEF [47.94 (31.33–65.10)% vs. 58.45 ± 14.14%] decreased and LV mass (124.02 ± 49.32 g vs. 96.14 ± 34.18 g) increased in the T2DM with CKD group (all *P* < 0.05).

**Fig. 1:**
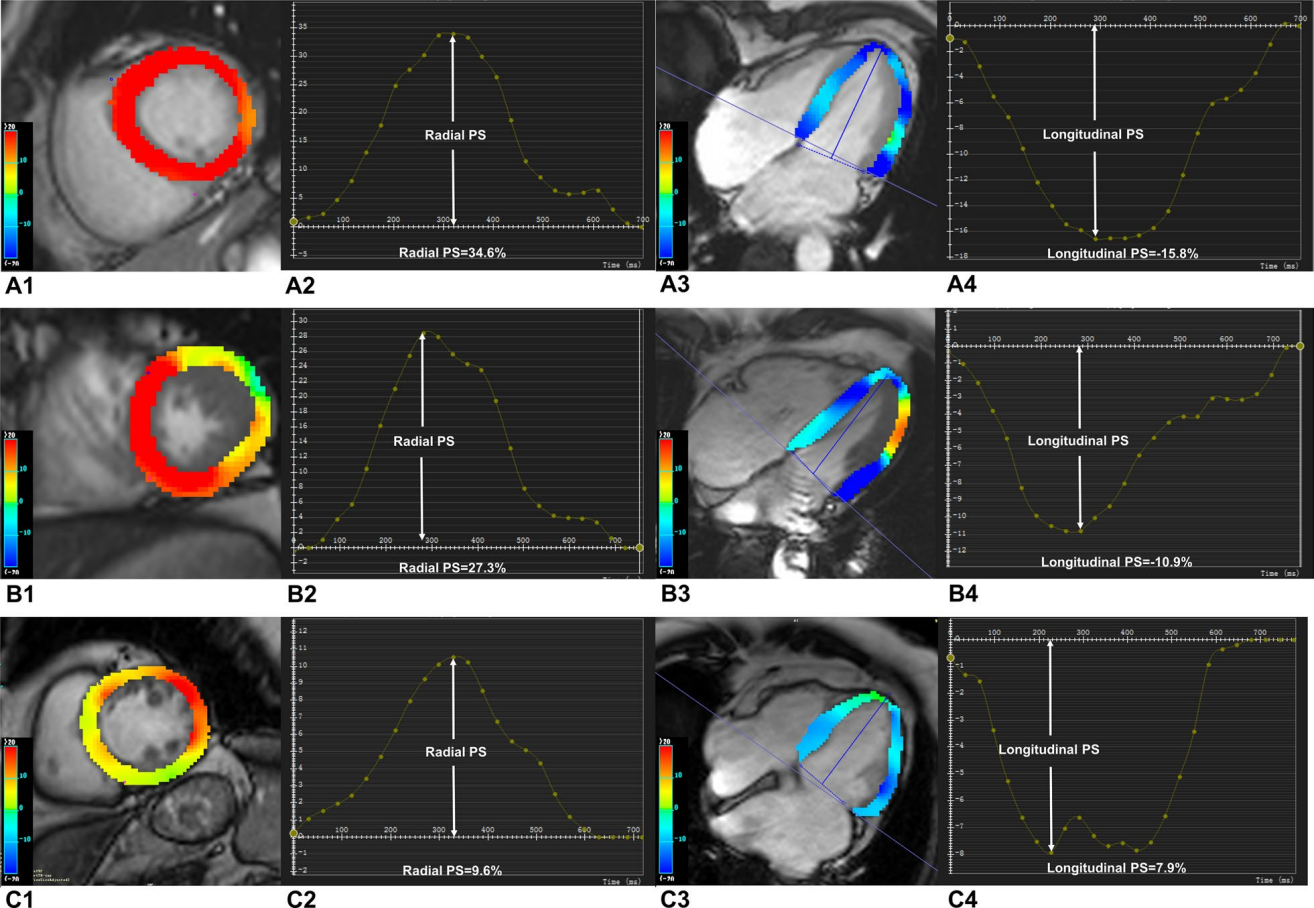
Representative CMR pseudocolor images at the end-systole and CMR-derived peak strain curves in a normal control, T2DM patient without CKD, and T2DM patient with CKD. **A1**–**C1** left ventricle pseudocolor images in short-axis; **A2**–**C2** LV global peak strain curve in radial direction; **A3**–**C3** left ventricle pseudocolor images in horizontal 4-chamber long-axis; **A4**, **B4**, **C4 **LV global peak strain curves in longitudinal direction

Regarding strain parameters, the magnitudes of radial (20.79 ± 12.02% vs. 38.42 ± 8.73%), circumferential (– 13.48 ± 6.16% vs. – 19.94 ± 2.71%), and longitudinal (– 8.35 ± 3.39% vs. 14.82 ± 3.33%) PS in T2DM with CKD group were remarkably lower than that in normal group (all *P* < 0.05). Furthermore, compared to T2DM patients without CKD, T2DM patients with CKD had markedly lower magnitudes of radial (20.79 ± 12.02% vs. 30.82 ± 10.48%), circumferential (– 13.48 ± 6.16% vs. – 19.37 ± 5.48%), and longitudinal (– 8.35 ± 3.39% vs. – 13.49 ± 3.78%) PS (all *P* < 0.05). Concurrently, the circumferential and longitudinal PDSR in T2DM patients with CKD were also lower than that in normal individuals and T2DM patients without CKD (Table 2). Figure 1 showed representative CMR cine images and CMR-derived peak strain curves in a normal control, T2DM patient without CKD, and T2DM patient with CKD.

### Association between the magnitude of LV PS and kidney function indices in T2DM patients

**Fig. 2:**
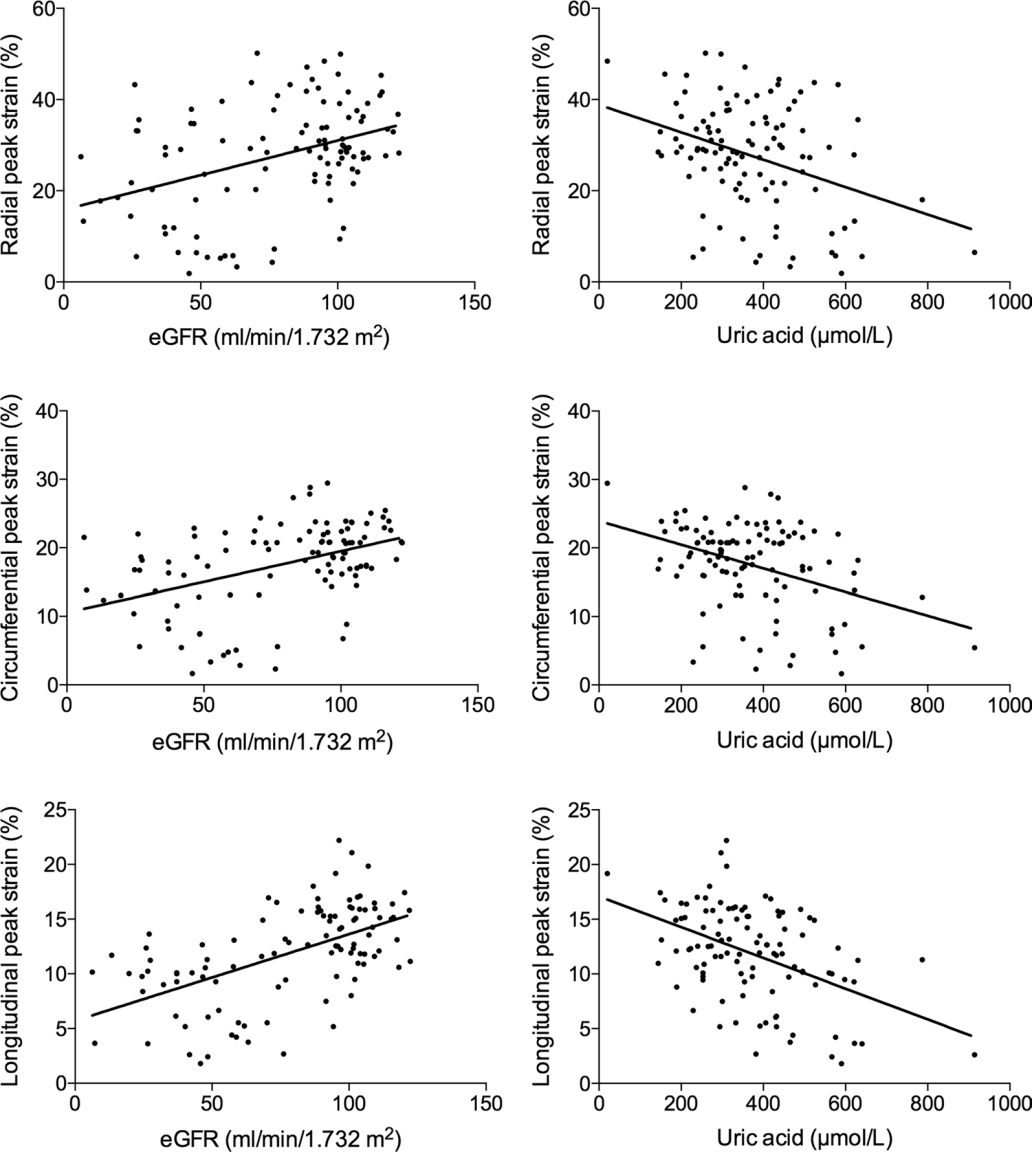
Linear regression analysis between the magnitude of LV peak strain (radial, circumferential, and longitudinal) and eGFR or uric acid. eGFR, estimated glomerular filtration rate

The correlations between LV PS and clinical indices are presented in Table 3. Within all diabetic patients in this cohort, Spearman correlation analysis showed that eGFR had a positive correlation with the magnitude of PS (R = radial, 0.392; circumferential, 0.436; longitudinal, 0.556, all *P* < 0.001) and that uric acid had a negative correlation with the magnitude of PS (R = radial, – 0.361; circumferential, – 0.391; longitudinal, – 0.460) (all *P* < 0.001) (Fig. 2). In addition, the creatinine and urea were also significantly associated with PS (all *P* < 0.05). There was no significant correlation between other clinical parameters and PS (all *P* > 0.05). Multivariable stepwise linear regression analysis indicated that eGFR (β = radial, 0.314; circumferential, 0.292; longitudinal, 0.500) and uric acid (β = radial, – 0.239; circumferential, – 0.211; longitudinal, – 0.238) (all *P* < 0.05) (Table 4) were independently associated with the magnitude of PS.

**Table 3:** Univariable correlations between the magnitude of LV peak strain and clinical indices in diabetic patients

	Radial PS		Circumferential PS					Longitudinal PS							
	R	*P*	R				*P*	R				*P*			
Creatinine	– 0.255	0.009	– 0.211				0.031	– 0.350				< 0.001			
eGFR	0.392	< 0.001	0.436				< 0.001	0.556				< 0.001			
Urea	– 0.269	0.005	– 0.259				0.008	– 0.343				< 0.001			
Uric acid	– 0.361	< 0.001	– 0.391				< 0.001	– 0.460				< 0.001			
Diabetes duration	0.099	0.313	0.022				0.825	– 0.084				0.395			
Fasting plasma glucose	0.059	0.552	– 0.005				0.961	0.101				0.305			
HbA1c	– 0.110	0.263	– 0.138				0.159	– 0.078				0.427			
Total cholesterol	– 0.156	0.112	– 0.101				0.307	– 0.052				0.601			
Triglycerides	– 0.016	0.869	– 0.023				0.815	0.076				0.439			

*eGFR *estimated Glomerular Filtration Rate, *HbA1c *glycated hemoglobin

**Table 4:** Multivariable linear analysis of LV strains in all diabetic patients adjusted for age, gender, BMI, systolic blood pressure, rest heart rate, eGFR, urea, and uric acid

	Radial PS			Circumferential PS			Longitudinal PS		
	β	*P*	R^2^	β	*P*	R^2^	β	*P*	R^2^
Age	0.197	0.027	0.226	0.201	0.019	0.282	0.032	0.677	0.419
Gender (Male)	– 0.035	0.698		– 0.105	0.222		– 0.063	0.416	
BMI	0.176	0.047		0.161	0.059		0.035	0.653	
Systolic blood pressure	0.076	0.405		0.138	0.121		0.305	< 0.001	
Rest heart rate	– 0.101	0.305		– 0.182	0.057		– 0.127	0.135	
eGFR	0.314	0.003		0.292	0.006		0.500	< 0.001	
Urea	– 0.010	0.927		– 0.002	0.069		0.046	0.618	
Uric acid	– 0.239	0.022		– 0.211	0.038		– 0.238	0.008	

*P < 0.05;

*BMI *body mass index, *eGFR *estimated glomerular filtration rate

### The reproducibility of CMR tissue tracking to access LV PS

The reproducibility of LV PS was considered excellent. The ICC values in the intraobserver analysis were 0.942 (95% confidence interval [CI] 0.908–0.965), 0.950 (95% CI 0.920–0.970), and 0.978 (95% CI 0.965–0.987) for radial, circumferential, and longitudinal PS, respectively. The ICC values in the interobserver analysis were 0.845 (95% confidence interval [CI] 0.766–0.903), 0.863 (95% CI 0.920–0.970), and 0.937 (95% CI 0.901–0.960) for radial, circumferential, and longitudinal PS, respectively.

## Discussion

The present study investigated the characteristics of LV function and strain in T2DM patients with or without CKD using CMR imaging. We verified the occurrence of decreased LV strain in T2DM patients compared to normal individuals. Furthermore, kidney dysfunction was proven to aggravate the deterioration of LV strain in T2DM patients with CKD. Finally, the eGFR and uric acid were independently associated with LV PS in radial, circumferential, and longitudinal directions.

In T2DM patients without CKD, our data revealed a reduction in radial PS, radial PSSR, radial PDSR, and circumferential PDSR compared to normal individuals, but no significant difference in LVEF was observed between these two groups, which was consistent with previous strain data regarding myocardial mechanics in DM [18–22]. The first 3-dimensional CMR strain study showed that longitudinal and circumferential PS as well as peak strain rates are impaired in type 2 diabetes with normal LVEF [20]. Similarly, Vukomanovic et al. reported that global longitudinal and circumferential strains are significantly reduced in diabetic participants and affect all LV myocardial layers [21]. Thus, the reduction of LV strain in T2DM patients may precede overt LVEF.

The underlying mechanism of reduced strain is still incompletely understood. It was demonstrated that hyperglycemia enhances fatty acid metabolism, suppresses glucose oxidation, and modifies intracellular signaling and leads to interstitial and perivascular fibrosis, contributing to reduction in ventricular compliance [22, 23]. Cao et al. [24] demonstrated that T2DM patients exhibited significantly increased mean native T1 values and ECVs of the LV compared with controls. Moreover, microvascular endothelial inflammation, rarefaction and perivascular collagen, and end-products deposition results in microvascular dysfunction, affecting cardiac contractility. Liu et al. [7] demonstrated an inverse relationship between time to maximum signal intensity and radial PS as well as a positive relationship between time to maximum signal intensity and longitudinal PS. These studies indicated that diabetes has an adverse effect on subclinical LV systolic dysfunction and myocardial perfusion.

Although CKD is also a common comorbidity of DM, studies on the additive effect of kidney dysfunction on cardiac function and deformation in T2DM are scarce. Thus, we conducted the present study and revealed that the magnitude of radial, circumferential, and longitudinal PS was markedly lower in T2DM patients with CKD than in both normal individuals and T2DM patients without CKD. Moreover, the LVEDV, LVESV, LV mass and LV remodelling index were markedly enlarged in T2DM patients with CKD compared to both normal individuals and T2DM patients without CKD. Taken together, these observations suggested that LV function progressively decreases in diabetic patients with CKD and is accompanied by left ventricle enlargement and hypertrophy.

In addition, we identified that the eGFR and uric acid were independently associated with the magnitude of LV PS in the radial, circumferential, and longitudinal directions, indicating that the magnitude of PS may gradually decrease with the deterioration of eGFR and the accumulation of uric acid. Thus, T2DM patients with high uric acid and low eGFR levels have an increased risk of myocardial strain deterioration. In CKD patients, mechanisms including hemodynamic instability, activation of the neuroendocrine system, oxidative stress, and anaemia, contribute to myocardial injury and resulting in poor cardiac outcomes during the process of chronic kidney dysfunction [25–27]. It can be inferred that the renal dysfunction had an additive adverse effect on LV in T2DM patients, contributing to myocardial abnormal deformation and cardiac contractile disability. This is partly explained by the activation of Rho-associated coiled-coil-containing protein kinase which is reported as an essential element in the development of atherosclerosis, hypertrophy of cardiomyocyte, cardiac fibrosis and cell death in T2DM patients with CKD [28, 29]. Therefore, focusing on changes in LV strain may facilitate the clinical management of T2DM patients with CKD to mitigate CVD risk and improve outcomes.

## Limitations

There are several potential limitations in our study. First, this was a single centre study. The sample size is relevant small but adequately powered to demonstrate the additive effects of kidney dysfunction on left ventricular function and strain in type 2 diabetes mellitus patients. A larger cohort or multicentre study with pooled data is required in the future. Second, long-term follow-up data related to the impact of LV strain changes on cardiovascular events was not obtained, and further longitudinal studies are required to investigate the potential of LV strain for predicting cardiovascular outcomes in T2DM patients with CKD. Thirdly, subclinical coronary ischemic disease may not be excluded because the stress test was not performed. However, the clinical coronary artery disease was considered to be unlikely according to the evaluation of patients by clinical history, laboratory results, echocardiography and electrocardiography. Results could still reflect the additive effect of kidney dysfunction on diabetic cardiomyopathy after adjustment for other confounders including age, gender, BMI, systolic blood pressure, rest heart rate. Last, the fasting plasma glucose and lipid status parameters of the controls were not measured. However, we carefully checked the detailed medical history and medical examination reports of enrolment for the controls to ensure meeting the inclusion criteria.

## Conclusions

LV global strain is significantly compromised in T2DM patients, and kidney dysfunction may aggravate the deterioration of LV strain in diabetic patients. LV strain is positively associated with the estimated glomerular filtration rate and negatively associated with uric acid, which may be independent risk factors for predicting reduction of LV strain. Therefore, more attention should be paid to LV strain evaluation in T2DM patients with CKD.

## Data Availability

The datasets used and analyzed during the current study are available from the corresponding author on reasonable request.

## Abbreviations

DM: Diabetes mellitus
T2DM: Type 2 diabetes diabetes mellitus
CKD: Chronic kidney disease
CMR: Cardiac magnetic resonance
LV: Left ventricle
BMI: Body mass index
HDL: High density lipoprotein
LDL: Low density lipoprotein
eGFR: Estimated glomerular filtration rate
EDV: End-diastolic volume
ESV: End systolic volume
SV: Stroke volume
LVEF: Left ventricle ejection fraction
PS: Peak strain
PRS: Peak radial strain
PCS: Peak circumferential strain
PS: Peak strain
PSSR: Peak systolic strain rate
PDSR: Peak diastolic strain rate
ICC: Intraclass correlation coefficient
CI: Confidence interval

## References

[1] Cho NH, Shaw JE, Karuranga S, Huang Y, da Rocha Fernandes JD, Ohlrogge AW, Malanda B (2018). IDF Diabetes Atlas: Global estimates of diabetes prevalence for 2017 and projections for 2045. Diabetes Res Clin Pract.

[2] Thomas MC, Cooper ME, Zimmet P (2016). Changing epidemiology of type 2 diabetes mellitus and associated chronic kidney disease. Nat Rev Nephrol.

[3] Bramlage P, Lanzinger S, van Mark G, Hess E, Fahrner S, Heyer CHJ, Friebe M, Seufert J, Danne T, Holl RW (2019). Patient and disease characteristics of type-2 diabetes patients with or without chronic kidney disease: an analysis of the German DPV and DIVE databases. Cardiovasc Diabetol.

[4] Forbes JM, Cooper ME (2013). Mechanisms of diabetic complications. Physiol Rev.

[5] Gavara J, Rodriguez-Palomares JF, Valente F, Monmeneu JV, Lopez-Lereu MP, Bonanad C, Ferreira-Gonzalez I, Garcia Del Blanco B, Rodriguez-Garcia J, Mutuberria M, de Dios E, Rios-Navarro C, Perez-Sole N, Racugno P, Paya A, Minant G, Canoves J, Pellicer M, Lopez-Fornas FJ, Barrabes J, Evangelista A, Nunez J, Chorro FJ, Garcia-Dorado D, Bodi V (2018). Prognostic value of strain by tissue tracking cardiac magnetic resonance after ST-segment elevation myocardial infarction. JACC Cardiovasc Imaging.

[6] Pedrizzetti G, Claus P, Kilner PJ, Nagel E (2016). Principles of cardiovascular magnetic resonance feature tracking and echocardiographic speckle tracking for informed clinical use. J Cardiovasc Magn Resonance.

[7] Liu X, Yang ZG, Gao Y, Xie LJ, Jiang L, Hu BY, Diao KY, Shi K, Xu HY, Shen MT, Ren Y, Guo YK (2018). Left ventricular subclinical myocardial dysfunction in uncomplicated type 2 diabetes mellitus is associated with impaired myocardial perfusion: a contrast-enhanced cardiovascular magnetic resonance study. Cardiovasc Diabetol.

[8] Jiang L, Wang J, Liu X, Li ZL, Xia CC, Xie LJ, Gao Y, Shen MT, Han PL, Guo YK, Yang ZG (2020). The combined effects of cardiac geometry, microcirculation, and tissue characteristics on cardiac systolic and diastolic function in subclinical diabetes mellitus-related cardiomyopathy. Int J Cardiol.

[9] Li XM, Jiang L, Guo YK, Ren Y, Han PL, Peng LQ, Shi R, Yan WF, Yang ZG (2020). The additive effects of type 2 diabetes mellitus on left ventricular deformation and myocardial perfusion in essential hypertension: a 3.0 T cardiac magnetic resonance study. Cardiovasc Diabetol.

[10] Gao Y, Ren Y, Guo YK, Liu X, Xie LJ, Jiang L, Shen MT, Deng MY, Yang ZG (2020). Metabolic syndrome and myocardium steatosis in subclinical type 2 diabetes mellitus: a ^1^H-magnetic resonance spectroscopy study. Cardiovasc Diabetol.

[11] Chamberlain JJ, Rhinehart AS, Shaefer CF, Neuman A (2016). Diagnosis and management of diabetes: synopsis of the 2016 American Diabetes Association Standards of Medical Care in Diabetes. Ann Intern Med.

[12] Levey AS, Eckardt KU, Dorman NM, Christiansen SL, Cheung M, Jadoul M, Winkelmayer WC (2020). Nomenclature for kidney function and disease: executive summary and glossary from a kidney disease: improving global outcomes consensus conference. Clin Kidney J..

[13] Maron BJ, Towbin JA, Thiene G, Antzelevitch C, Corrado D, Arnett D, Moss AJ, Seidman CE, Young JB, American Heart Association; Council on Clinical Cardiology, Heart Failure and Transplantation Committee; Quality of Care and Outcomes Research and Functional Genomics and Translational Biology Interdisciplinary Working Groups; Council on Epidemiology and Prevention (2006). Contemporary definitions and classification of the cardiomyopathies: an American Heart Association Scientific Statement from the Council on Clinical Cardiology, Heart Failure and Transplantation Committee; Quality of Care and Outcomes Research and Functional Genomics and Translational Biology Interdisciplinary Working Groups; and Council on Epidemiology and Prevention. Circulation.

[14] Matsushita K, Mahmoodi BK, Woodward M, Emberson JR, Jafar TH, Jee SH, Polkinghorne KR, Shankar A, Smith DH, Tonelli M, Warnock DG, Wen CP, Coresh J, Gansevoort RT, Hemmelgarn BR, Levey AS (2012). Chronic Kidney Disease Prognosis Consortium. Comparison of risk prediction using the CKD-EPI equation and the MDRD study equation for estimated glomerular filtration rate. JAMA.

[15] Schulz-Menger J, Bluemke DA, Bremerich J, Flamm SD, Fogel MA, Friedrich MG, Kim RJ, von Knobelsdorff-Brenkenhoff F, Kramer CM, Pennell DJ, Plein S, Nagel E (2020). Standardized image interpretation and post-processing in cardiovascular magnetic resonance—2020 update: Society for Cardiovascular Magnetic Resonance (SCMR): Board of Trustees Task Force on Standardized Post-Processing. J Cardiovasc Magn Reson.

[16] Schlett CL, Lorbeer R, Arndt C, Auweter S, Machann J, Hetterich H, Linkohr B, Rathmann W, Peters A, Bamberg F (2018). Association between abdominal adiposity and subclinical measures of left-ventricular remodeling in diabetics, prediabetics and normal controls without history of cardiovascular disease as measured by magnetic resonance imaging: results from the KORA-FF4 Study. Cardiovasc Diabetol.

[17] Krishnasamy R, Hawley CM, Stanton T, Pascoe EM, Campbell KL, Rossi M, Petchey W, Tan KS, Beetham KS, Coombes JS, Leano R, Haluska BA, Isbel NM (2015). Left ventricular global longitudinal strain is associated with cardiovascular risk factors and arterial stiffness in chronic kidney disease. BMC Nephrol.

[18] Collier P, Phelan D, Klein A (2017). A test in context: myocardial strain measured by speckle-tracking echocardiography. J Am Coll Cardiol.

[19] Tadic M, Cuspidi C, Calicchio F, Grassi G, Mancia G (2020). Diabetic cardiomyopathy: how can cardiac magnetic resonance help?. Acta Diabetol.

[20] Fonseca CG, Dissanayake AM, Doughty RN, Whalley GA, Gamble GD, Cowan BR, Occleshaw CJ, Young AA (2004). Three-dimensional assessment of left ventricular systolic strain in patients with type 2 diabetes mellitus, diastolic dysfunction, and normal ejection fraction. Am J Cardiol.

[21] Vukomanovic V, Suzic-Lazic J, Celic V, Cuspidi C, Petrovic T, Grassi G, Tadic M (2019). The relationship between functional capacity and left ventricular strain in patients with uncomplicated type 2 diabetes. J Hypertens.

[22] T.Miki S, Yuda H, Kouzu T, Miura (2013). Diabetic cardiomyopathy: pathophysiology and clinical features. Heart Fail.

[23] Markus MRP, Rospleszcz S, Ittermann T, Baumeister SE, Schipf S, Siewert-Markus U, Lorbeer R, Storz C, Ptushkina V, Peters A, Meisinger C, Bamberg F, Nauck M, Bahls M, Völzke H, Felix SB, Bülow R, Rathmann W, Dörr M (2019). Glucose and insulin levels are associated with arterial stiffness and concentric remodeling of the heart. Cardiovasc Diabetol.

[24] Cao Y, Zeng W, Cui Y (2018). Increased myocardial extracellular volume assessed by cardiovascular magnetic resonance T1 mapping and its determinants in type 2 diabetes mellitus patients with normal myocardial systolic strain. Cardiovascular Diabetology.

[25] Odudu A, Eldehni MT, McCann GP, Horsfield MA, Breidthardt T, McIntyre CW (2016). Characterisation of cardiomyopathy by cardiac and aortic magnetic resonance in patients new to hemodialysis. Eur Radiol.

[26] Rutherford E, Talle MA, Mangion K, Bell E, Rauhalammi SM, Roditi G, McComb C, Radjenovic A, Welsh P, Woodward R, Struthers AD, Jardine AG, Patel RK, Berry C, Mark PB (2016). Defining myocardial tissue abnormalities in end-stage renal failure with cardiac magnetic resonance imaging using native T1 mapping. Kidney Int.

[27] Kluger AY, Tecson KM, Lee AY, Lerma EV, Rangaswami J, Lepor NE, Cobble ME, McCullough, Class effects of SGLT2 inhibitors on cardiorenal outcomes (2019). PACardiovasc Diabetol.

[28] Matoba K, Takeda Y, Nagai Y, Sekiguchi K, Yokota T, Utsunomiya K, Nishimura R (2020). The physiology, pathology, and therapeutic interventions for ROCK isoforms in diabetic kidney disease. Front Pharmacol.

[29] Shimizu T, Liao JK (2016). Rho kinases and cardiac remodeling. Circ J.

